# The Pathophysiology of Spontaneous Preterm Birth: Emerging Mechanisms Reviewed by the Preterm Birth International Collaborative

**DOI:** 10.1007/s43032-026-02108-5

**Published:** 2026-05-06

**Authors:** Emmeli Mikkelsen, Emmanuel Amabebe, Andrea Olmos-Ortiz, Emily Hamburg-Shields, Egle Bytautiene Prewit, Oksana Shynlova, Deena L. Gibbons, Sam Mesiano, Michael J. Taggart

**Affiliations:** 1 Department of Clinical Medicine, Aarhus University, Aarhus, Denmark; 2 Department of Obstetrics and Gynaecology, Aarhus University Hospital, Aarhus, Denmark; 3 Division of Basic Science and Translational Research, Department of Obstetrics and Gynecology, University of Texas Medical Branch at Galveston, Galveston, TX USA; 4 Division of Clinical Medicine, School of Medicine & Population Health, Faculty of Health, University of Sheffield, Sheffield, UK; 5 Immunobiochemistry Department, Instituto Nacional de Perinatología Isidro Espinosa de los Reyes, Mexico City, Mexico; 6 Department of Reproductive Biology, Case Western Reserve University, Cleveland, OH USA; 7 Department of Ob/Gyn, University Hospitals of Cleveland, Cleveland, OH USA; 8 Division of Reproductive Research, Department of Obstetrics and Gynecology, University of Texas Health San Antonio, San Antonio, TX USA; 9 Lunenfeld Tanenbaum Research Institute, Sinai Health System, Toronto, Canada; 10 Department of Obstetrics and Gynaecology, University of Toronto, Toronto, Canada; 11 Peter Gorer Department of Immunobiology, School of Immunology and Microbial Sciences, King’s College London, London, UK; 12 Biosciences Institute, Newcastle University, Newcastle, UK

**Keywords:** Parturition, Preterm birth, Pathophysiology, Inflammation, Biomarkers, Vaginal microbiome, Progesterone

## Abstract

Around 10% of global births are preterm (before 37 weeks of gestation), posing a significant challenge to maternal and neonatal health. Preterm infants face an increased risk of mortality and long-term health complications, impacting their survival and development across all life stages. Despite decades (~ 80 years) of research, effective methods to predict and prevent idiopathic or spontaneous preterm birth remain limited. Therefore, a deeper understanding of the pathophysiology of spontaneous preterm birth is warranted. This review explores some aspects of recent progress in unravelling the complex pathophysiology of both normal and preterm human birth. We present parturition as an inflammatory event, triggered by stressors affecting the uterine reproductive tissues (myometrium, decidua, and cervix), and involving multiple endocrine and paracrine pathways. These pathways, along with signals from fetal membrane senescence and the vaginal microbiome, contribute to labor induction. Proposed perspectives in parturition research include using mathematical modeling and machine learning (artificial intelligence) to map pregnancy trajectories and identify patient phenotypes associated with preterm birth risk. Additionally, incorporating preterm birth history into routine life course medical surveillance for affected individuals and their offspring is recommended. Finally, increased investment and prioritization from national funding bodies, along with greater support for international collaborations, are essential to identify the causes of preterm birth across multiple populations and develop new, effective treatments.

## Introduction

Globally, around 10% of births (~ 13.4 million annually) are preterm (before 37 completed weeks of gestation) [1]. This presents a major challenge, as neonates born preterm are at higher risk of mortality (~ 700,000 per year; 35% of all neonatal deaths) [2, 3], primarily due to functionally immature organ systems vital for survival outside the uterus. Furthermore, survivors of preterm birth (PTB) face an increased risk of lifelong disabilities.

Every decade, the “Born Too Soon” Global Action Report on Preterm Birth measures the extent of the problem, the progress made, and proposes solutions to reduce PTB rates [4]. Despite over 80 years of research, however, effective methods to predict and prevent spontaneous PTB (sPTB) remain limited. Additionally, while improved access to antenatal healthcare has been made, there has been no measurable change in PTB rates over the past decade [1, 4].

In 2015, the United Nations’ Sustainable Development Goal set a target to reduce neonatal mortality to no more than 12 per 1,000 live births by 2030. Achieving this goal, even over a longer timeframe, requires a dramatic decrease in sPTB rates. Therefore, a deeper understanding of the pathophysiology of sPTB is crucial.

This review, authored by members of the Preterm Birth International Collaborative (PREBIC), explores recent progress in understanding the complex (patho)physiology underlying the process and timing of both normal and preterm human birth, including advances in early monitoring of sPTB risk, prevention and treatment options in women presenting with preterm labor (PTL). We hypothesize that this understanding is rooted in the knowledge of hormonal signals (both endocrine and paracrine) from maternal and fetal tissues that influence the labor state of the gravid uterus. Several key concepts will be discussed, including parturition as an inflammatory event triggered by stressors on the uterine effector tissues (myometrium, decidua, and cervix), which leads to labor induction through multiple endocrine and paracrine pathways. These pathways are influenced by upstream signals from placental senescence and the vaginal microbiome. Furthermore, emerging topics in parturition research, such as systems biology, mathematical modeling, and machine learning (artificial intelligence), are discussed for their potential to map pregnancy trajectories and uncover the patient phenotypes underlying PTB risk. Additionally, the life course implications for both affected individuals and their offspring are explored. 

## Emerging Mechanisms in the Pathophysiology of Spontaneous Preterm Birth

### Parturition – An Inflammatory Event

Data from clinical and animal studies show that parturition is a localized physiologic inflammatory process [5]. The maternal immune system undergoes major transformation and activation during pregnancy, likely impacted by the constantly changing hormonal environment. These changes are critical to maintain pregnancy and prevent a rejection of the semi-allogeneic fetus [6].

**Fig. 1:**
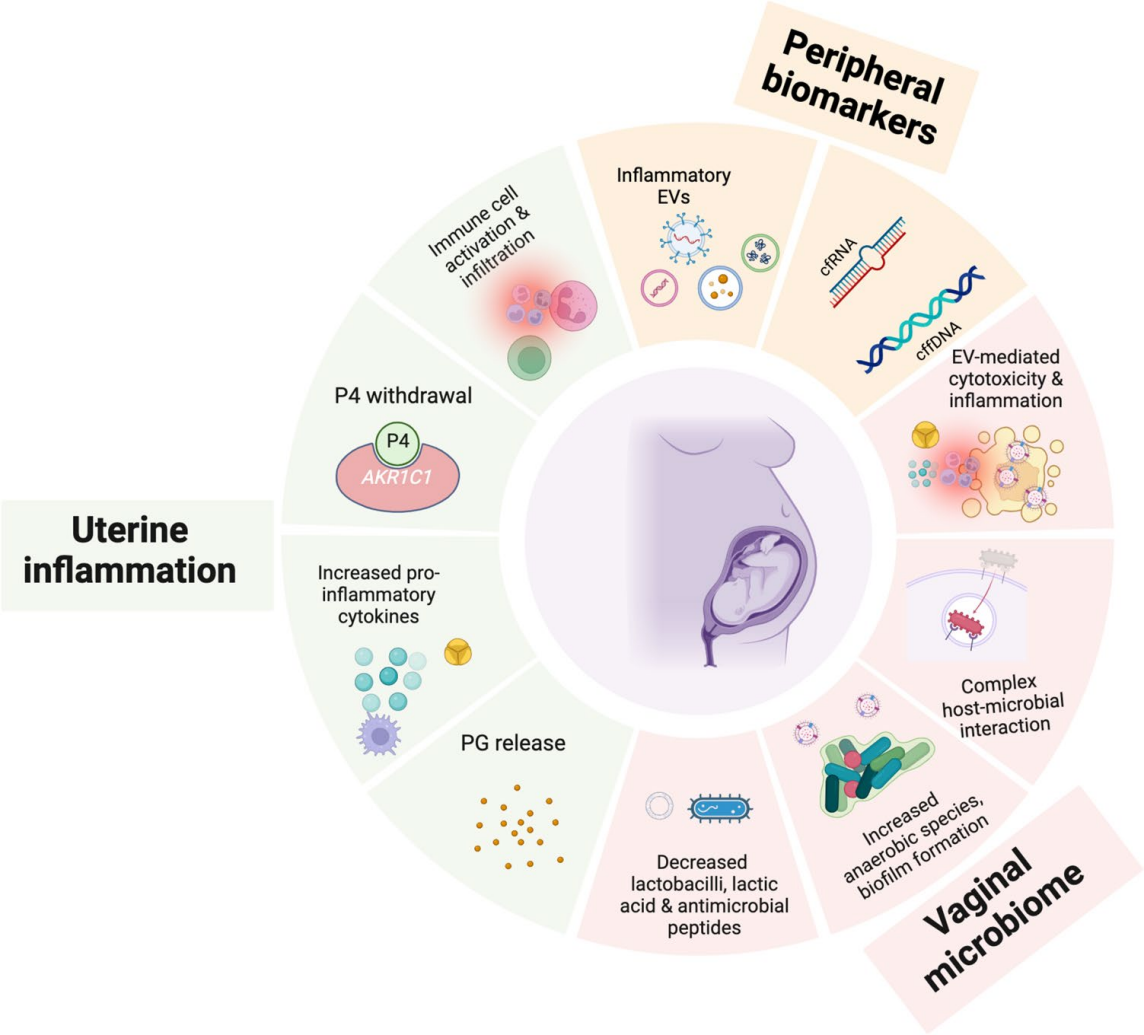
Emerging mechanisms in the pathophysiology of spontaneous preterm birth. *AKR1C1* aldoketo-reductase 1C1; *EV* extracellular vesicles; *PG* prostaglandin; *P4* progesterone. *Created with BioRender.com*

One of the first recognized gestation-related changes in the peripheral blood is an increase in leukocyte count [7], in particular neutrophilia of gestation [8–11]. During pregnancy, there is an increase in the concentrations of acute-phase proteins in the plasma, as well as elevated numbers of monocytes and neutrophils [12, 13]. Several groups have reported that circulating leukocytes are activated in pregnant women without clinical signs of infection [14–16]. The preparation for term parturition involves a physiological inflammatory process coordinated by both fetal and maternal uterine tissues. This process is characterized by an upregulation in the secretion of pro-inflammatory cytokines and chemokines by fetal membranes (amnion-chorion), and uterine tissues, including cervix, decidua (endometrium of pregnancy) and smooth muscle (myometrium) capable of activating maternal peripheral leukocytes (Fig. 1) [17]. Chemokine receptors are constitutively expressed on maternal peripheral immune cells [18].

Pro-inflammatory cytokines activate maternal peripheral leukocytes circulating in the blood and the vascular endothelium of the uterus, facilitating their infiltration into uterine reproductive tissues (Fig. 1) [19]. At the end of gestation, even in the absence of infection, the density of leukocytes in the uterine tissues (cervix, myometrium and decidua) increases, reaching the highest level at around term labor [19]. Accumulating leukocytes promote cervical ripening (dilatation and effacement), membrane/decidual activation and myometrial contractions, expulsion of the neonate and placenta [20]. Infiltration of leukocytes (i.e., macrophages) into the decidua precedes infiltration into the myometrium suggesting sequential temporal and spatial activation in response to increased cytokine secretion [21, 22]. The decidua serves as a dynamic site for chemokine synthesis, playing a crucial role in attracting various immune cells, such as neutrophils, natural killer cells, dendritic cells, macrophages, and T lymphocytes. These decidual leukocytes actively contribute to creating an immunogenic-tolerant environment to prevent the rejection of the fetal semi-allograft [23]. Additionally, they play vital roles in blastocyst implantation, regulation of placental angiogenesis, remodeling of uterine spiral arteries, and phagocytosis of trophoblastic apoptotic bodies and senescent fetal cells [24].

Following infiltration, maternal peripheral monocytes differentiate into tissue macrophages. Studies in mice have shown that one-third of immune cells in the decidua are macrophages, and their numbers double prior to labor [25]. In term decidual tissues, anti-inflammatory M2-like macrophages outnumber pro-inflammatory M1-like macrophages, while during spontaneous term labor and PTL, macrophages undergo M1-like polarization [26]. In complicated pregnancies, the M1/M2 ratio is altered [27]. Various studies have observed the presence of neutrophils, macrophages, and lymphocytes (NK and T-cells) within the human decidua and fetal membranes before and during labor [5, 28]. Studies in mouse pregnancy show that macrophage depletion can prevent infection-induced PTB and cervical remodeling [29, 30]. Moreover, recent reports using a murine model of in utero sterile inflammation induced by the intra-amniotic injection of the alarmin HMGB1, show beneficial effects of adoptive transfer of M2-like macrophages capable of preventing PTB and improving newborn health [31, 32]. Uterine-infiltrated leukocytes secrete labor mediators, such as uterotonins capable of promoting synchronous contractions of the myometrium, and express matrix metalloproteinases (MMPs) involved in fetal membrane rupture [33] and cervical ripening [34, 35]. There is also a possibility that immune cells, such as neutrophils, monocytes, or T-cells, play a crucial role in postpartum uterine involution, decidual shedding, and post-delivery healing processes [36].

The underlying inflammatory mechanisms appear to be similar for term labor and PTL. It was suggested that premature activation of the maternal immune system (either by infection or by other risk factors) can trigger premature cervical ripening, myometrial and/or decidual activation (i.e. cytokine secretion causing leukocyte influx) and PTL leading to the delivery of a preterm infant [22, 37, 38]. Numerous research efforts have been made to understand changes in these cell populations that may predispose pregnant individuals to PTB. A significant challenge lies in clinically monitoring changes in decidual populations throughout gestation, especially during the second and early third trimesters. Healthy term and PTB decidua have been analyzed in the human, whereas mice have been employed to explore events from mid-pregnancy onward [39–41]. It is essential to note that immune interactions in animal models may not entirely reflect the immunological changes occurring in the human decidua. Investigating the frequency and phenotypic profile of decidual leukocytes could provide insights into vulnerability to ascending infections from the cervico-vaginal tract, a closely related factor for sPTB.

Numerous studies were undertaken in the past decade to reveal molecular events responsible for term labor initiation to predict targets for drug development and novel therapies that would prevent PTB in high-risk pregnancies. Leukocytes are an active component of the maternal immune system; therefore, they can provide relatively accessible means to interrupt the inflammatory pathway leading to inflammation and labor initiation. Inhibitors of chemokine signaling that can simultaneously block multiple molecular pathways (i.e. broad spectrum chemokine inhibitors, BSCIs) have recently been developed [42]. In a wide range of animal models, BSCIs improve different diseases such as allergic asthma, surgical adhesion formation, rheumatoid arthritis, HIV replication, and endometriosis [43–49]. Recent studies have demonstrated that BSCI prevents PTB in mice [41] and non-human primates [50]. In particular, BSCI decreases in vivo infection-induced uterine inflammation by inhibiting chemokine-mediated monocyte and neutrophil infiltration into the mouse myometrium [41], as well as preventing cervical ripening [51]. More importantly, in pregnant monkeys (*Macaca nemestrina*) BSCIs blocked pre-term myometrial contractions and decreased maternal plasma chemokine levels [50], while in vitro it prevents trans-endothelial migration of human leukocytes [52], and macrophage-myocyte communication [53].

In addition to the decidua, the placenta serves as a reservoir for leukocytes originating from the fetal circulation, localized within the villous tissue. These leukocytes play a crucial role in trophoblast surveillance, and there are documented instances highlighting their involvement in combating infections [54]. Recent research explores novel areas, such as understanding how fetal immune cells within the placenta contribute to immune tolerance while ensuring protection against infections. Another avenue of investigation explores the influence of bacterial extracellular vesicles from the placenta and its interplay with immune cells [55]. The advent of single-cell technologies has facilitated the analysis of individual immune cell populations within the placenta, offering a more intricate comprehension of cell–cell interactions and their respective functions.

### Paracrine Mediators of Inflammation and Parturition

In most species, the physiology of parturition is dominated by endocrine signals involving hormones that relay information from the fetus to indicate its readiness for life as a neonate [56]. This is exemplified in sheep [57]. In this species, parturition is triggered by increased cortisol production by the fetal hypothalamic–pituitary–adrenal (HPA) axis which promotes maturation of organ systems in the fetus and induces maternal progesterone (P4) withdrawal in the that triggers parturition [58]. This endocrine mechanism is not apparent in human parturition. Instead, paracrine signals between trophoblast cells and decidual stromal cells appear to play central roles in the maintenance of pregnancy and the initiation of parturition. This occurs mainly in the chorion-decidua interface (CDI) lining the uterine wall. This tissue microenvironment is adjacent to myometrium and cervix and as such factors produced in the CDI may readily diffuse to those tissues to control the engine (myometrium) and getaway (cervix) for parturition. Paracrine interactions between chorion trophoblast cells and decidual stromal cells also involve resident immune cells in the CDI that are critical for the establishment and maintenance of pregnancy; with aberrations associated with adverse pregnancy outcomes such as preeclampsia and PTB [59, 60]. A key factor is P4 produced by chorion trophoblast cells in high amounts throughout pregnancy. P4 diffuses to decidual stromal cells and myometrial cells where it interacts with cognate P4 receptors (PRs) to affect the expression of genes whose products generally promote myometrial quiescence and cervix closure [61–64]. Via decidual stromal cells, P4 also may modulate the activity of resident immune cells to induce an immune state that is tolerant of the allogeneic fetal cells. A leading hypothesis is that this pro-pregnancy state ends when P4 signaling is lost and this leads to immune cell activation and inflammation in the CDI. Prostaglandins (PGs) produced by the inflammation (i.e., activated immune cells) would diffuse to the myometrium to stimulate contractions and to the cervix to promote softening and dilation (Fig. 1) [65, 66]. A key characteristic of this model is that inflammation may induce P4 withdrawal in decidual stromal cells and uterine myocytes by inducing expression of the aldoketo-reductase 1C1 (*AKR1C1*) that converts P4 to an inactive form thus preventing its interaction with PRs (Fig. 1) [67]. This localized P4 withdrawal leads to tissue level inflammation that transitions the myometrium and cervix to the labor state. Blocking *AKR1C1* activity or the use of Selective Progesterone Receptor Modulators resistant to *AKR1C1* degradation is a plausible approach to decrease the risk for inflammation-induced parturition by preventing loss of the P4/PR block to parturition [51, 68, 69].

Emerging evidence from Lintao et al. (2024) identifies a critical role for chorion trophoblast cells (CTCs) in maintaining immune quiescence at the chorion-decidua interface (CDi), a key maternal–fetal boundary implicated in the timing of labor. Using a human “CDi-on-chip” co-culture model, the study demonstrates that CTCs suppress local inflammation and immune cell infiltration via paracrine signaling involving locally produced progesterone, the non-classical progesterone receptor PGRMC2, and the immunomodulatory molecule HLA-G. CTCs do not express the nuclear progesterone receptors PR-A or PR-B, which mediate canonical genomic progesterone genomic signaling. Despite this, CTCs actively synthesize progesterone and participate in regulating local immune environments – raising the question of how they might respond to the hormone. CTCs express PGRMC2, which regulates the epithelial identity of these cells and promotes immune tolerance by supporting HLA-G expression. Knockout of PGRMC2 or HLA-G leads to mesenchymal transition, increased expression of inflammatory cytokines, and loss of barrier function at the CDi. It is proposed that these changes facilitate decidual immune cell migration and cytokine activation – hallmarks of labor initiation [70]. Therefore, a critical, non-genomic progesterone-regulated mechanism in the fetal membranes appears to complement systemic endocrine cues and may serve as an upstream trigger for both term and preterm labor. These findings highlight the need to expand models of progesterone withdrawal to include spatially distinct paracrine mechanisms at the fetal-maternal interface.

### The Vaginal Microbiome and Spontaneous Preterm Birth

The vaginal ecological niche comprises host epithelial and mucosal cells in a dynamic and sensitive mutualistic relationship with the microbiota [71]. The composition of a “healthy” vaginal microbiota (VMB) varies by race and ethnicity and is influenced by factors such as age, menstrual cycle, pregnancy, parity, sexual intercourse, contraceptive and antibiotic use, diet, smoking, drug use, obesity, stress, hygiene, and host genetics [71–78]. Generally, vaginal homeostasis is propagated by dominance of lactobacilli that produce lactic acid (to acidify the milieu) and antimicrobial peptides to the detriment of pathogenic microorganisms. Hence, *Lactobacillus* depletion and/or overgrowth of anaerobic species leads to dysbiosis and increased risk of vaginal infections including bacterial vaginosis (BV) – a risk factor for sPTB (Fig. 1) [71–73, 79]. Although the normal vaginal microbiota is dominated by *Lactobacillus sp.* regardless of ethno-racial or geographic affiliations [80], asymptomatic women of African ancestry living in low-middle income countries (LMICs) harbor VMB different from African women living in developed countries as well as women of other races [81–84]. The VMB of asymptomatic African women can have less than 40% *Lactobacillus* dominance [82, 85], and is often dominated by *Lactobacillus iners* co-existing with high abundance of *Gardnerella vaginalis*, *Atopobium vaginae*, and *Prevotella sp.* [77, 82, 84, 86–88] compared to their white counterparts that harbor more of *L. crispatus, L. gasseri,* and *L. jensenii* [80, 81, 89]. A similarly high prevalence of *L. iners* is reported in Asian women, where *L. iners* was identified in 77% of pregnant women in an Indian study population [90]. Again, *L. iners* was frequently isolated from women with normal, intermediate or BV microbiota, whereas *L. crispatus* (present in all the study participants) and *L. jensenii* decreased significantly with the transition to intermediate and BV microbiota. Additionally, 68% of healthy and 89% of BV-infected non-pregnant women had *L. iners*-dominated microbiota in a Chinese study population, and *L. iners* negatively correlated with *L. gasseri* [91]. Meanwhile, it was reported that asymptomatic African American and Hispanic women in North America were more likely to harbor VMB comprising diverse bacterial species typically seen in BV [80].

Normal pregnancy is associated with a more stable, less diverse lactobacilliary VMB [92–96], with a relatively higher abundance of *L. crispatus, L. gasseri, L. jensenii,* and *L. vaginalis*, and lower abundance of 22 other non-Lactobacillus species [97, 98]. Whilst a direct causal relationship is yet to be established, women with a more diverse [99, 100], less stable (low-lactobacilli) VMB are at greater risk of PTB compared to women with a more stable, *L. crispatus-*dominated microbiota, which is often associated with female reproductive health and positive outcomes including term deliveries [71, 97, 98, 101–120]. The low-lactobacilli VMB permits dominance of anaerobes and BV-associated bacteria such as *Gardnerella*, *Prevotella, Atopobium, Ureaplasma*, *Mobiluncus*, *Megasphaera*, *Dialister,* and *Sneathia*, that promote inflammation and adverse outcomes including PPROM and sPTB [100, 101, 110–112, 114, 121–128], while *L. crispatus* is anti-inflammatory, and promotes optimal vaginal health [71, 72, 106–109, 129–131] and favorable reproductive outcomes including protection against sPTB [71, 77, 99, 103, 104, 110–112, 116, 123, 124, 126, 132]. However, in a high-risk cohort where 32% of the preterm women were of African ancestry, the association between vaginal dysbiosis (community state type, CST IV) and PTB was not confirmed, but *L. iners* dominance was a risk factor for PTB [116]. Another study conducted in a Canadian cohort was also unable to detect VMB biomarkers (CSTs) early in pregnancy that could predict PTB [92]. Moreover, in some women of African ancestry, low lactobacilli and high *Gardnerella* abundances do not pose significant risk for PTB [110, 133]. Furthermore, some Caucasian women with *L. jensenii* dominance have increased risk of PTB [98], whereas *L. iners* dominance is common among women of African [116, 133] and Asian [116, 134] ancestry regardless of birth outcomes. Therefore, the evidence that changes in VMB increases risk of sPTB is still debatable, and context-specific or population-dependent description and interpretation of the association is advisable [110, 123].

The health-promoting and potentially harmful activities of the vaginal commensal bacteria described above are also propagated by the extracellular vesicles (EV) they secrete. For instance *L. crispatus* and *L. gasseri* EVs protect human cells from HIV-1 infection [135], whereas *G. vaginalis* EVs taken up by vaginal epithelial cells induced vaginolysin-mediated cytotoxity and release of interleukin (IL)−8 (Fig. 1) [136, 137]. Despite the deleterious actions of harmful bacterial species on gestation, the association between vaginal dysbiosis or infection and PTB is still debatable due to other intrinsic and extrinsic modifiable and non-modifiable factors [138]. There is also the question of what constitutes a “normal” VMB [75]. These controversies are exacerbated by the failure of treatment of genitourinary tract infection with antibiotics alone to reduce PTB, or prolong pregnancy, even when initiated earlier in gestation [138, 139]. Furthermore, in the context of chronic disease, antibiotic use during pregnancy may increase the risk of PTB [140]. Though the reason for the lack of efficacy of antibiotic treatment to reduce PTB is largely unknown, the VMB appears to be a potentially modifiable antenatal risk factor for PTB. Additionally, variations in host immune responses are still a potential culprit that requires more exploration. This is because both VMB and local [111] and systemic [88, 141–147] immune responses modulate the risk of sPTB.

Ultimately, PTB risk is determined by a complex host-microbial immune interaction rather than prevalence of a single microbial taxon or community state type [139]. Early diagnosis of abnormal VMB in pregnant individuals at risk of infectious or inflammatory PTB can be achieved by using advanced molecular techniques including microbiome assessment for organism identification, multiplex quantitative PCR testing for identification and quantification of specific organisms [148], and various tests for antimicrobial resistance [138]. This could guide treatment strategies combining antibiotics effective against BV-related organisms, probiotics or anti-inflammatory agents that can readily cross the placental barrier, which may reduce the incidence of PTB [71, 149–151].

### Biomarkers for Spontaneous Preterm Birth

A biomarker is an objective, quantifiable substance/structure/process in the body or its products that provides insight into the physiology or pathophysiology of a healthy or disease condition [152]. Therefore, by identification of potential biomarkers for sPTB, new interventions and treatments can be discovered [153]. In this section, we discuss new areas of research that have shown great promise to generate novel biomarkers for sPTB with adequate exploration.

#### Extracellular Vesicles

EVs are nano-sized, membrane-bound, non-replicating vesicles secreted by all cells as a means of communications with other nearby or distant cells and tissues in both physiological and pathological conditions [154–156]. EVs are a snapshot of the present state of their cells of origin, and based on their size, biogenesis, cargo, and function, EVs are classified into exosomes (30–160 nm), microvesicles (MVs) (100–1000 nm), and apoptotic bodies (~ 50–5,000 nm) [155, 157, 158]. EVs protect their cargo (nucleic acids, proteins, lipids, and metabolites) from degradation and can deliver them to target tissues to elicit a response [158]. Hence, EVs act as paracrine signalers providing a potential source of circulating biomarkers, indicators of physiologic state and disease existence/progression, and as vectors for targeted therapies [156, 158].

EVs are found in several biological fluids including peripheral blood, saliva, urine, amniotic fluid, vaginal fluid, tracheal fluid, tears, breast milk, and umbilical cord blood [158, 159]. EVs participate in initiating normal labor at term and in the pathogenesis of PTB [155, 156, 160]. Infection, inflammation, oxidative stress, or environmental pollutants can initiate PTB by inducing premature release of inflammatory exosomes that are trafficked across the placenta in both directions (Fig. 1) [156, 157, 161–163]. This inflammatory exosomal signaling is mediated by nuclear factor kappa-light-chain-enhancer of activated B cells (NF-κB), IL-6, IL-8, PGE_2_, and p38 mitogen-activated protein kinases (p38 MAPK) [157].

Both human and bacterial EVs have been identified in gestational tissues including the placenta and amniotic cavity [55, 158, 164, 165]. The bacterial EVs which can emanate from the gut [164], oral, vaginal, respiratory tract, integumentary microbiota or external environment [55, 165] can be taken up by human cells, and both bacterial and human EVs can exchange their cargos [158]. Although still understudied, this can help bacterial EVs, and their cargos circumvent host immune clearance and perpetuate immune responses that prepare (fine-tune) the fetal immune system for survival *ex-utero* [55, 164, 165]. However, in some unresolved instances, the EVs and/or their cargos can induce feto-maternal immune intolerance that disrupts the progression of pregnancy leading to adverse pregnancy outcomes including sPTB [158]. Details of how human and bacterial EVs exchange cargos is extensively discussed by Amabebe et al*.* [158].

Consistent with the foregoing observations, EV-derived inflammatory proteins, miRNAs, and lipids not only differ significantly between term and preterm women but also predict placental dysfunction. Exosome biomarkers for PTL and PTB have been identified in maternal plasma across different gestational time points [157, 166–169]. First trimester “circulating microparticles” contained a unique panel of proteins (F13A, FBLN1, IC1, ITIH2, and LCAT) [170] that predicted PTB (≤ 35 weeks) with an area under the curve (AUC) of 0.74 (95% confidence interval (CI) 0.63–0.81) [171]. The AUC slightly improved to 0.77 (95% CI 0.61–0.90) with a separate panel of proteins (IC1, LCAT, TRFE, and ITIH4) as markers to predict high-risk status in nulliparous women at 10–12 weeks of gestation [171]. In women presenting with symptoms of PTL between 24–34 weeks, plasma EVs containing miRNAs were identified as biomarkers for PTL, and indicators of pathological changes in the placenta during PTL [172]. This study was followed by the identification of a miRNA profile targeting TGF-β, p53, and glucocorticoid receptor signaling pathways in exosomes from maternal plasma that were altered according to gestational age and believed to represent a biomolecular "fingerprint" of pregnancy progression [169]. Another study identified miR-612, which is associated with apoptosis in tumor cells and regulation of NF-κB inflammatory pathway, mediating PTL/PPROM pathogenesis was increased in plasma-derived small EVs of preterm-delivered women [167].

Menon et al*.* also reported dramatic changes in the proteome of circulating placental EVs expressing membrane-bound placental alkaline phosphatase (PLAP) that could reveal underlying biological mechanisms that lead to early parturition and stratify women at risk of PTB [168]. Proteins associated with coagulation/complement activation were downregulated in preterm-delivered women while those associated with epithelial mesenchymal transition pathways were upregulated in term-delivered women [168]. Furthermore, lipidomic analysis of plasma-derived MVs at 12–24 weeks of gestation revealed a panel of five lipids (PS (34:0), PS (O-42:0), PI (O-36:1), C24 (OH) sulfatide and PE (O-33:0)) that predicted PTB (AUC = 0.87 (95% CI 0.87–0.94), sensitivity = 100% and specificity ~ 71.2%). Using a pseudotargeted lipidomics approach, one lipid of the panel (PS (34:0)) was validated in an additional cohort (AUC = 0.71 (95% CI 0.60–0.82), sensitivity = 63.4% and specificity = 76.2%) [166]. However, only one lipid in plasma and none in exosome had an AUC > 0.8. Therefore, the authors reported that lipids in MVs represent the most effective predictors of PTB with sufficient sensitivity and specificity compared to lipids in plasma and exosomes [166].

More recently, first trimester detection of altered M1 and Th17 responses within urinary EVs predicted PTB with MCP-1 (> 174 pg/mL) exhibiting a sensitivity of 71.9% and a specificity of 64.6%, while a combination of MCP-1 (> 174 pg/mL) and IFNγ (> 8.7 pg/mL) showed a higher sensitivity (84.6%) but a moderate specificity of 66.7% for predicting PTB [173]. Subsequently, urinary EVs from women with term deliveries suppressed M1 and Th17 differentiation compared to those from preterm-delivered women that induced a significantly higher production of IL-8 and TNFα cytokines through higher expression of chromatin modification at histone 3 lysine 4 trimethylation (H3K4me3) [173]. The ability of urinary EVs from term-delivered women to modulate altered M1 and Th17 polarization associated with better T-cell regulatory differentiation could be a potential preventive intervention for subsequent PTB [173]. Moreover, in mice, after vaginal infection with *E. coli*, intravenous injections of anti-inflammatory IL-10 encapsulated in exosomes delayed PTB by reducing fetomaternal uterine immune cell inflammation [150].

These reports along with the ability of EVs to carry an assortment of pro-inflammatory genetic materials including cell-free DNA and RNA [158, 159] indicate great promise for the utility of EVs as early predictors for PTB. However, researchers should define the EV isolation methods and particle size employed in the assay analysis as different nomenclatures are used to describe particles analyzed in different studies [157].

#### Cell-Free DNA

Cell-free DNA (cfDNA) is fetal DNA of placental or fetal membrane origin circulating freely in maternal blood [174]. cfDNA increases with gestation due to physiological remodeling of placental trophoblast and breakdown of the placental barrier in anticipation of labor [175–178]. Hence, cfDNA is employed as a biomarker for prenatal diagnosis of pregnancy complications including PTB (Fig. 1) [175, 179]. Currently, the main indication for cfDNA screening is fetal aneuploidy [180]. In mouse models with additional immune impairment, intraperitoneal injection of fetal DNA or CpG induces Toll-like Receptor (TLR)−9 and NF-κB-mediated inflammatory responses in uterine tissues that increase IL-6 expression which could trigger PTL/PTB [179, 181, 182]. However, the ability of cfDNA to activate pro-inflammatory responses through this DNA sensing mechanism that involves TLR9 and Stimulator of Interferon Genes (STING) pathway to cause sPTB has not been established [179].

Meanwhile, clinical studies report that increased cfDNA in second and third trimester is associated with greater risk of PTL/sPTB in high-risk pregnancies and nulliparous women at low risk [178, 183–186]. cfDNA ≥ 95th percentile at 14–20 weeks and 25 weeks predicted risk of PTB < 34 weeks with an AUC of 0.65 ((95% CI 0.48–0.81), sensitivity = 33% and specificity = 96%) [183] and of 0.711 (95% CI 0.51–0.92) [178], respectively. However, other studies have reported no association [180, 187–193]. For example, while second trimester cfDNA levels > 95th percentile could increase the risk of PTB by 16-fold [178, 183], similar levels at first trimester or around the beginning of second trimester were not associated with increased risk in women screened for aneuploidy [187, 191]. Instead, low levels < 10th percentile were associated with early PTB [194]. Overall, out of ten studies that evaluated the association of cfDNA (measured between first trimester and 25 weeks of gestation) and PTB in asymptomatic women, along with prenatal testing for fetal anueploides, majority (60%) did not identify an association, while 40% observed an increase in cfDNA in women destined to deliver preterm [179]. In addition to these ten studies, one study indicated an association [184], and two studies reported no association between increased cfDNA and sPTB [180, 187].

These contrasting results are attributed to heterogeneity in sampling, indications for screening, quantification methods, etc. [179, 180, 188], and have contributed to the low performance and clinical utility of cfDNA as an early predictor of sPTB. Elevated cfDNA as seen in normal labor may merely be associated with loss of uterine quiescence rather than the pathophysiological causes of sPTB [188, 195]. Nonetheless, maternal cfDNA may increase prior to PTB induced by systemic inflammation (not intra-amniotic inflammation) [196]. Furthermore, the rise in cfDNA observed in individuals who eventually deliver preterm and the potential pro-inflammatory action of cfDNA indicate a potential mechanistic role in the pathogenesis of sPTB [179]. Therefore, there is need for more comprehensive longitudinal studies investigating PTB-associated temporal changes in cfDNA levels in pregnancies with or without fetal aneuploidy. In the meant time, as the quest for a more useful non-invasive test for sPTB continues, the field appears to have advanced towards cfRNA.

#### Cell-Free RNA

Like cfDNA, cell-free RNA (cfRNA) released from fetal, fetal membrane and placental tissues can be detected in maternal plasma and employed as a non-invasive diagnostic and prognostic test for sPTB (Fig. 1) [197]. Differentially expressed regulatory RNA in placentas of preterm infants are detectable in maternal circulation as cfRNA [198, 199]. cfRNA transcripts that include placental and fetal signals can accurately track pregnancy progression independent of clinical factors such as maternal age, race and body mass index (BMI) [198]. This cfRNA profile accurately predicted gestational age to about 2 weeks (14.7 days) similar to second trimester ultrasound and better than third trimester ultrasound, providing an alternative test to date pregnancies of unknown gestational age [198, 199].

Recently, strongly expressed mRNA, ncRNA, snoRNA, and snRNA were observed in maternal plasma of preterm-delivered women compared to the placenta [200]. Subsequently, the cfRNA *ARHGEF28* showed significant predictive ability for risk of whole PTB (AUC = 0.99) and late PTB (AUC = 0.99) [200]. Increased levels of another cfRNA, *TNFSF4,* was observed in women with PTL, and negatively correlated with gestational age at delivery [201]. Moreover, *TNFSF4* was postulated to be a novel non-invasive biomarker for PTL (AUC = 0.76 (95% CI 0.499–1.0), and an indicator of immune crosstalk at the fetomaternal interface that initiate labor at term or preterm [201]. Many other cfRNAs of maternal as well as fetal origin including *HMGB1* and *TLR4* that are associated with inflammatory PTL were also reported [201]. For example, *TLR4*, *ARG2*, and *TNFSF4* were observed from maternal peripheral blood myeloid cells [201]. PTL-associated *TLR4* and *EIF2AK2* were also overexpressed in inflammatory macrophages from placenta and fetal lungs respectively [201, 202].

Additionally, different cfRNAs associated with collagen or extracellular matrix (ECM) degradation and remodeling predicted risk of early (< 35 weeks) sPTB with an AUC of 0.80 (95% CI 0.72–0.87), sensitivity = 76% and specificity = 72%). Whereas genes associated with insulin-like growth factor transport and amino-acid metabolism predicted risk of very early sPTB (< 25 weeks) (AUC = 0.76 (95% CI 0.63–0.87), sensitivity = 64% and specificity = 80%) [203]. Although still requiring validation, detection of cfRNAs associated with ECM degradation at second trimester may identify individuals at risk of premature cervical remodeling, a screening that could be employed at a similar gestation as the ultrasound to measure cervical length of high-risk women [203]. On the other hand, insulin-like growth factor binding protein (IGFBP) is associated with fetal growth restriction (FGR) and abnormal placentation and raised in cord blood of extremely preterm infants (IGFBP1) [204] or upregulated in whole blood RNA preceding PPROM (IGFBP2) [205]. Inflammatory cfRNA of placental origin have also been detected in amniotic fluid and predicted sPTB within 24 h of amniocentesis with an AUC of 0.81 [206]. The upregulated genes are involved in myeloid leukocyte activation, complement activation, TLR signaling, B-cell-mediated immunity, NK and T-cell-mediated cytotoxicity [206]. Advancing toward a diagnostic test for PTB, a lateral flow test that detects endogenous concentrations of miR-150-5p (AUC = 0.97) in maternal blood from the 12th week of gestation has been reported [207]. miR-150-5p is associated with the NF-κB pathway that is linked to the initiation of labor [208] and was previously predictive of PTB from as early as 12 weeks (AUC = 0.87, specificity = 64%) [209]. In another instance, maternal blood cfRNAs associated with placental development (*RAB27B*), pro-platelet basic protein (*PPBP*) and genes that influence pregnancy through inflammation (*DAPPI, RGS18*), labor (*CLCN3*), and development *MOB1B* were part of a model that predicted PTB with an AUC of 0.81 better than mass spectroscopy (insulin binding protein 4 and sex hormone binding globulin ratio, IBP4/SHBG), and positive predictive value of 80%, compared to cervical length (17%) and fetal fibronectin measurements (21%) [197]. The reliability or clinical applicability of these non-invasive blood tests still require validation in large clinical trials. Meanwhile, the current evidence has improved our understanding of the mechanisms of sPTB and creates a promising opportunity for more targeted therapeutics and effective interventions.

#### Other Biomarkers

Apart from the aforementioned biomarkers, other commercially available biochemical tests are employed in different settings albeit with varying limitations. They include: 1) the mid-trimester vaginal fluid swab-based quantitative fetal fibronectin test [210–212]; 2) the Actim® Partus test that detects phosphorylated insulin-like growth factor binding protein-1 (phIGFBP-1) from 22 weeks of gestation, albeit with high negative predictive value [213–215]; 3) the PreTRM™ test, which measures the log IBP4/SHBG ratio by proteomics between 18 and 20^+6^ weeks of gestation in blood (AUC 0.75) [216–218]; and 4) the PartoSure test that detects placental alpha microglobulin-1 (PAMG1) in cervicovaginal fluid obtained by vaginal swab [219]. These biomarkers are employed in symptomatic women with threatened PTL as well as asymptomatic high-risk women. Additionally, a systematic review of 149 PTB biomarker studies that employed genomics, transcriptomics, proteomics and metabolomics techniques identified molecules that mediate infection-inflammation pathway such as IL-6, TNF, TLR4 and prostaglandins as the commonly reported biomarkers of PTB [220]. Maternal alpha fetoprotein and C-reactive protein have also shown good diagnostic accuracy in identifying individuals at risk of sPTB [221, 222]. These tests have good predictive performance as a “rule-out” test to identify individuals that are not at risk for PTB [223]. However, they do not identify the majority of patients who eventually deliver preterm in low-risk populations who have normal cervix and no previous history of PTB [223]. Interestingly, more recent research show that mid-trimester cervicovaginal glutamate (AUC 0.72) and a combination of glutamate, acetate and D-lactate (AUC 0.82) have good predictive performance for sPTB in asymptomatic low risk population [224], while acetate alone predicts imminent sPTB in individuals with threatened PTL [225–227]. The predictive performances of these metabolites improve when combined with quantitative fetal fibronectin and cervical length measurements [224–227]. Because PTB is a multifactorial syndrome of maternal, fetal, or by risk factors and pathophysiological pathways involving both, there is critical need to design biomarker discovery and management interventions based on early, mid, and late trimester specific markers of both fetal and maternal origin that identify the underlying cause(s) [223].

## Next Horizons in Understanding the Pathophysiology of Spontaneous Preterm Birth

### Clarity of Patient Phenotypes

The PTB syndrome has been conceptualized as a final common pathway that results from heterogeneous pregnancy complications as well as iatrogenic influences. To organize the distinct presentations of PTB is challenged by an enormous degree of clinical heterogeneity. Commonly used classification systems such as gestational age at birth, birth weight, antecedent event leading to PTB, and pathologic phenotypes vary in implementation in both the clinical and scientific realms [228]. Even the lower gestational age limit for viable prematurity varies significantly between study cohorts and in epidemiologic descriptors, in part because the lower limit of neonatal viability is highly dependent upon birth location and local resources. Many studies have dichotomized PTB into spontaneous and non-spontaneous (or iatrogenic or provider-initiated) PTB. Non-spontaneous PTB can be further differentiated by the clinical indication for PTB. sPTB can similarly be divided into PTL (cervical dilation and contractions with intact membranes) versus preterm prelabor rupture of membranes (PPROM).

While these systems have supplied the framework for insights and interventions in prematurity research, their simplicity is an inherent limitation. For example, the discrepancy between clinical trials of hydroxyprogesterone caproate for prevention of sPTB [229, 230] has popularly been attributed to clinical and demographic differences in the two study populations. The lack of implementation of a comprehensive PTB classification scheme by both clinicians and researchers is a barrier to robust investigation of the diverse pathologic pathways that lead to prematurity. Stringent classification of PTB phenotypes and risk profiles is also essential for identifying discrete cohorts of patients in whom a therapeutic strategy can be tested and demonstrate efficacy (following the model in which cervical cerclage has been demonstrated to be beneficial in patients with sonographic short cervix) [231].

In contemporary prematurity research, comprehensive classification strategies have been proposed [232–235], but their utilization in clinical and translational studies has been limited [236]. These strategies attempt to incorporate information that can be assessed prospectively, and which may address the etiology of the PTB (i.e. presence of suspected placental abruption, maternal comorbid conditions) and information such as placental histology which is available retrospectively and has greater objectivity than the clinical impression at the time of the initial patient presentation. Realization of benefits from a universal classification of PTB will require commitment to a common systematic approach that is practical for both bench and bedside investigators.

### Mathematical Modeling

Contractions of the uterus during labor, at term or preterm, are regulated by episodic electrical action potentials exciting myometrial smooth muscle cells of the organ. Understanding the biophysical processes underlying myometrial electrical excitation at the single cell, multicellular tissue and organ levels is key to informing improved strategies for the detection and tocolytic treatment of pregnancies complicated by sPTB. Computational (mathematical) modelling approaches in cardiac physiology and disease have led the way for decades in integrating such information from different spatiotemporal settings and research modalities to enable quantitative predictions that have been utilized for further scientific research and clinical diagnosis and treatment [237]. For example, there were over 250 publications on cardiac computational modelling in 2022 alone [237].

**Fig. 2:**
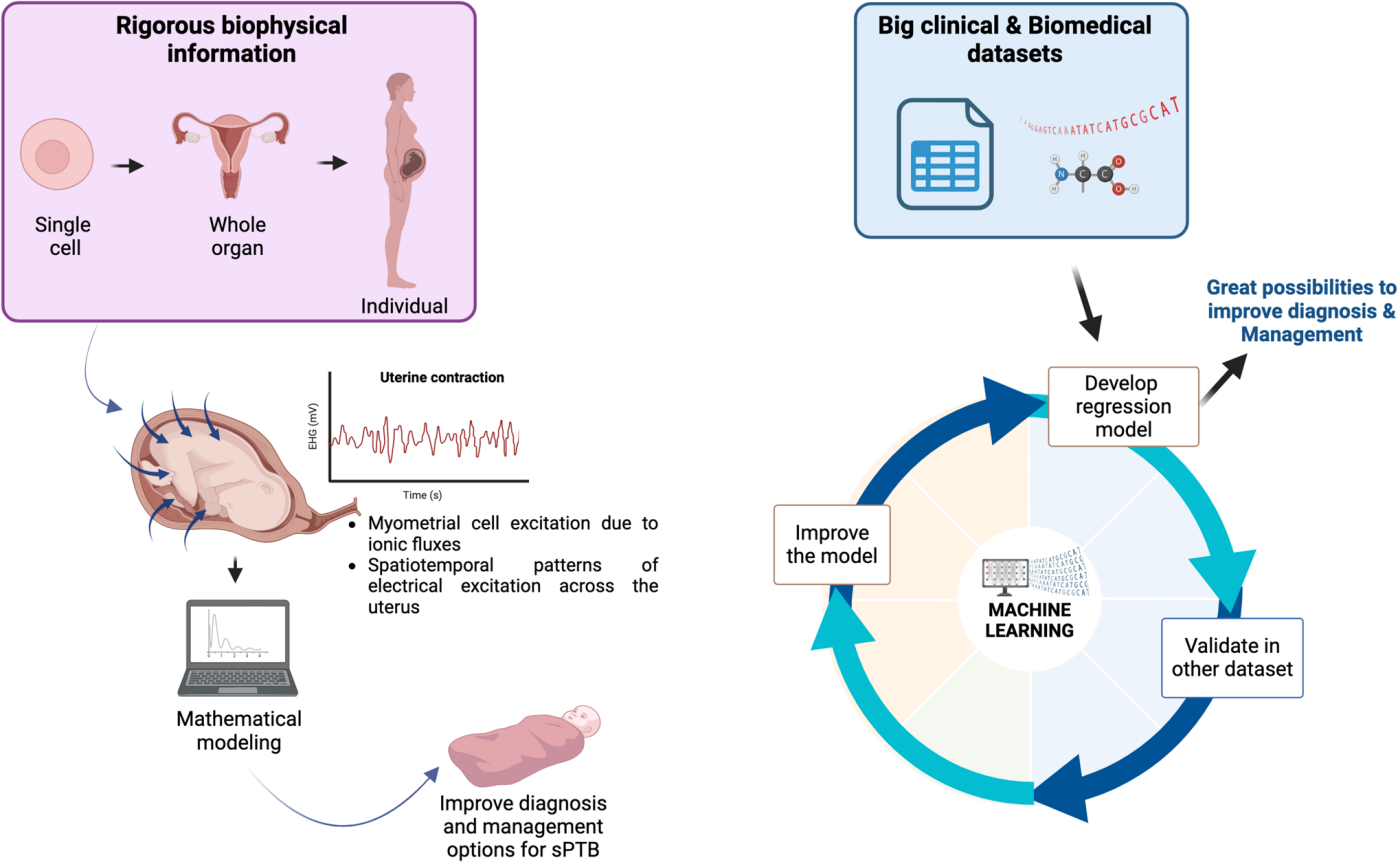
Mathematical modeling and machine learning to improve prediction, diagnosis, and management of spontaneous preterm birth. *EHG* electrohysterography. *Created with BioRender.com*

This indicates the scale of opportunity for computational modelling to fill significant knowledge gaps of relevance to understanding multicellular processes of uterine activation in normal labor. This is important for the development of well-informed artificial intelligence approaches (see below) to better identify what has gone awry in PTB and perform in silico tests of potential interventions to correct matters. Although this is an area under-utilized at present, notable progress has been made in the last decade or so in establishing quantitative descriptions of 1) ionic fluxes contributing to myometrial cell excitation [238–241] and 2) spatiotemporal patterns of electrical excitation across uterine tissue (Fig. 2) [242–245]. Moreover, recent advances in technical feasibility of recording electrohysterographic signals of uterine excitability with arrays of surface electrodes [246], and improved mathematical interpretation of the spread of such signals [247] offers diagnostic promise. For computational modelling approaches to reach their full potential in improving diagnosis of, and treatment options for sPTB, there needs to be considerable investment in the collection of rigorous biophysical information at all scales (single cell to whole organ to individual clinical parameters) (Fig. 2). Engagement with the continued development of machine learning/artificial intelligence tools (see section below) should assist in the integration of such complex data purposed towards understanding normal and PTL.

### Machine Learning (Artificial Intelligence)

With prematurity being a worldwide health issue, artificial intelligence (AI) could provide insightful information for solving this problem. The etiology of PTB is multifactorial, and AI provides the means of prediction for complex tasks. The number of studies using machine- or deep-learning to examine PTB has increased markedly in the past several years. Most studies using mathematical algorithms provide outputs using the input data and given rules, while AI produces rules and patterns using output and input data. In other words, mathematical algorithms could only explore limited data sets. In comparison, the AI explores and learns from an unlimited number of datasets. Then, the results are tested in validation datasets to determine the accuracy using the area under the receiver-operating characteristic curve and regression models (Fig. 2). This measurement reflects the quality and accuracy of the AI model.

Identifying risk factors is one area where AI could be applied to predict and detect PTB. Such a study was recently published and demonstrated that the consistency of the vaginal microbiome could predict PTB and early PTB [248]. In an analysis of PTB by trimester, the results of this study confirmed *Lactobacillus* species were negatively associated with PTB. However, the *Lactobacillus jensenii*-like phylotype was positively associated with PTB when present in the second trimester. Two distinct *Lactobacillus* were more prevalent with PTB when found in the third semester. Interestingly, the last two findings are contrary to the broad understanding that Lactobacillus is beneficial for PTB [248].

AI has been employed to predict PTB by analyzing various plasma and amniotic fluid biomarkers and using cervical parameters and clinical and epidemiological data [249]. In 2022, the systematic review of studies investigating the prediction of PTB using AI found that studies using metabolic panels and electrohysterogram images demonstrated the best accuracy [249]. The same systematic review acknowledged that studies should be performed on big data samples [249]. As such, an AI study used electronic medical records of 35,382 women with documented deliveries [250]. Models trained on only billing codes have promising potential to predict PTB, and they outperformed models trained on only clinical risk factors. Moreover, results were the same when models based on billing codes were examined on an external, independent cohort [250].

In addition to prediction, AI could assist in personalized treatment strategies for PTB by determining personal risk factors based on history, genetics, and other relevant factors [249, 250]. For example, using a combination of recordings from wearable devices and AI methods, it was shown that changes in physical activities and sleep are significantly associated with prematurity [251]. Further studies revealed that sleepers and movers have a 52% reduced risk of PTB. Pregnant women who sleep and move less have a 44% risk of delivering early [252].

Despite its promise, AI should be applied carefully to use reliable datasets for AI training, addressing biases and ethical considerations.

In summary, continuing advances in AI, applied to expanding and rigorously curated datasets (e.g. see section below), can improve the prediction, diagnosis, and treatment of PTB.

### Life Course Implications for Affected Individuals and Offspring

**Fig. 3:**
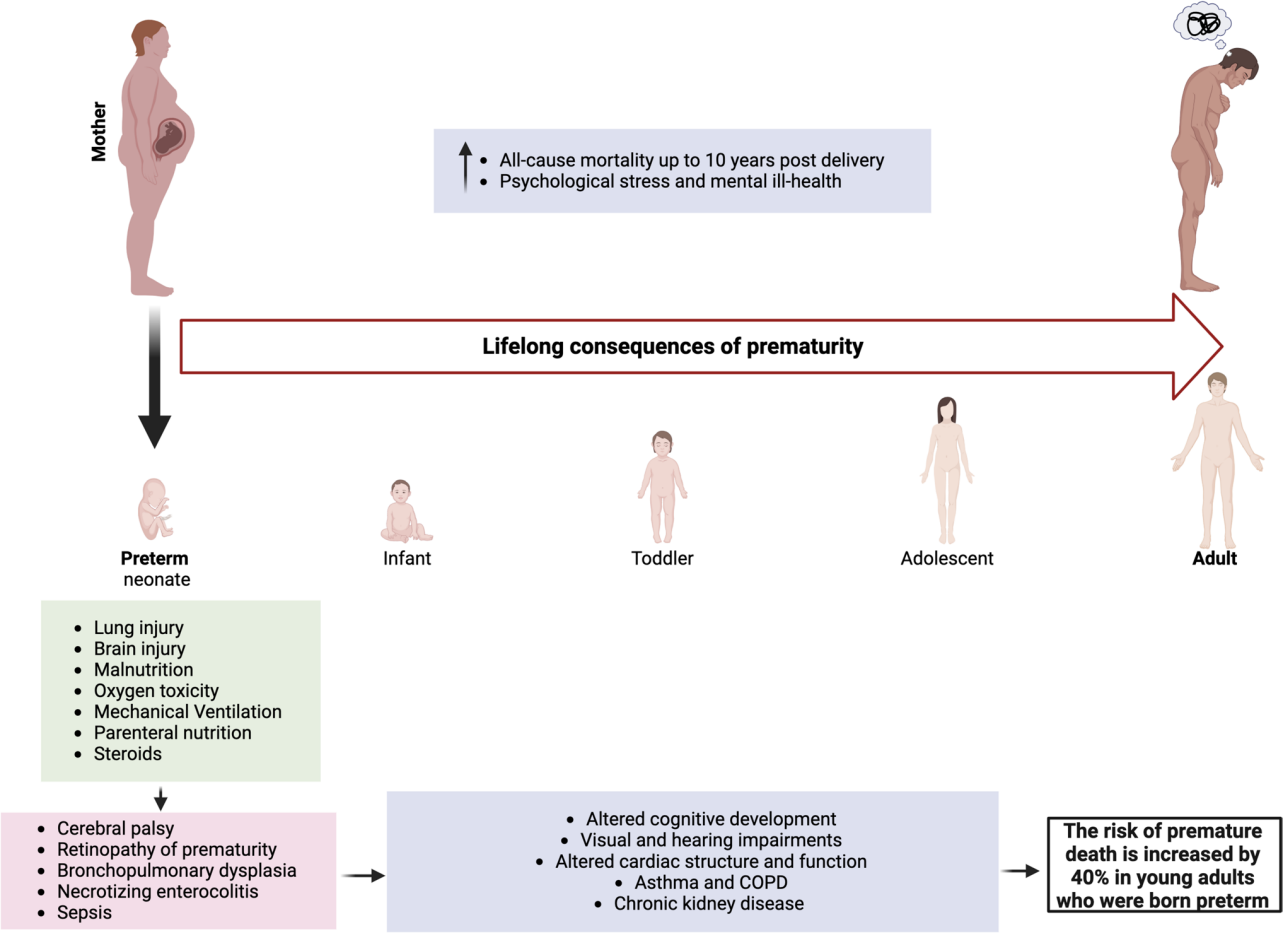
Lifelong consequences of preterm birth for affected individuals and surviving offspring. *COPD* chronic obstructive pulmonary disease. *Created with BioRender.com*

The mortality statistics for babies born preterm are stark, as are the immediate risks of ill-health for survivors [4]. As the causes of sPTB are many and varied, so is the outcome for the infant with respect to the longer-term health implications of being born prematurely. It is important to also give prominence to the increasing evidence of lifelong risks to health and wellbeing of being born prematurely as these are considerable [253–255] and that even by adolescence and young adulthood complex multimorbidities may be evident (Fig. 3) [256, 257]. Post-birth, a premature infant is confronted with drastically different exposures such as hospital-specific microbes, drugs, and changes in oxygen levels, which may play a role in the infant’s future health [258]. Short term complications directly associated with PTB, particularly bronchopulmonary dysplasia (BPD), necrotizing enterocolitis (NEC), retinopathy of prematurity (ROP) and sepsis are known to lead to longer term problems. Objectively, this is not surprising: preterm neonates begin their extra-uterine life from a very different starting point of organ maturation and systems physiology than those delivered after a full uncomplicated gestation. The impact of this can be exemplified by considering four organ systems: the brain, heart, kidneys, and lungs (Fig. 3).

sPTB has a stronger negative association with infant cognitive development in comparison to small for gestational age or low birth weight infants [259]. Often preterm infants demonstrate alterations across neurobehavioral function prior to neonatal intensive care unit (NICU) discharge validating that developmental delays in preterm infants are present early and not just emerging later in childhood [260].

Ultrasound and magnetic resonance imaging studies of adolescent subjects born preterm have indicated a number of alterations in cardiac structure and function compared to peers born at term [261]. This is consistent with emerging data from Scandinavian patient registry studies that a major risk factor for early-onset heart failure, in the absence of other confounders, is being born preterm [262, 263].

Interrogation of similar datasets has recently revealed a strong association of the incidence of later-life asthma and chronic obstructive pulmonary disease and PTB [264–266], perhaps related to structural differences (small airways) in the preterm lung as well as other immune/microbiome changes associated with PTB. Despite the observations that preterm immune systems appear to ‘catch up’ with that of a term infant [267, 268], the risk of rehospitalization due to infections during childhood is inversely correlated with gestational age [269]. Whilst this has been associated with reduced transplacental transfer of maternal antibodies in preterm infants, recent data suggests that the most functional antibodies are still transferred [270]. Interestingly, whilst asthma risk is elevated, allergic sensitization risk is decreased, highlighting that untangling the direct effects of PTB on future life is difficult and should be an area of ongoing research [271]. PTB is also associated with an increased risk of later-life chronic kidney disease likely to be related, in part, to impaired nephrogenesis by the shortened period in utero [272, 273].

Under-appreciated too are the potential long-term detrimental health outcomes of individuals who experienced pregnancies resulting in PTB (Fig. 3). Crump et al*.* recently interrogated data from the Swedish Medical Birth Register to ascertain the incidence of PTB among 2 million pregnant individuals and their mortality outcomes in a 44-year follow-up period [274]. All-cause mortality up to 10 years post-delivery was increased in parturients who had delivered preterm with the hazard ratio being greatest for those who had delivered between 28–33 weeks of gestation. Leading contributory causes included cardiovascular and respiratory disease, diabetes, and cancer. It is also notable that affected individuals have a higher risk of psychological distress and mental ill-health [275, 276]. Of relevance here too is that individuals with multimorbidity at the time of conception have an elevated risk of preterm delivery [277].

## Conclusion and Future Perspectives

There is a pressing need to emphasize that PTB is not a syndrome confined to the peripartum period. It presents significant risks of ill-health to individuals with a history of PTB and their surviving offspring throughout the life course. This highlights the need for continued clinical surveillance of both parturients and offspring affected by PTB, to ensure an optimal health trajectory. This surveillance should include the routine recording of 1) gestational age at birth, 2) maternal pregnancy outcomes, including gestational length in all medical records, and 3) continuous evaluation of diagnostic, preventive, and management strategies for PTB, both locally and globally.

We conclude this review with a recommendation for substantial increase in investment and prioritization from national funding bodies, along with enhanced facilities for international collaboration, in the study of pregnancy and PTB. The 2030 Sustainable Development Goals of the United Nations underscore the urgency and scope of this task if we are to definitively identify the causes of, and develop new, effective treatment options for, PTB. The research concepts emerging from this review present promising avenues for the next five years to address these critical aims.

## Data Availability

Not applicable.
